# Functional signatures of the gut microbiome in middle-aged regular runners: insights from a metagenomic study

**DOI:** 10.3389/fphys.2026.1826138

**Published:** 2026-07-10

**Authors:** Xu Ai, Ruijie Liu, Yongling Lv, Li Chen, Rui Duan, Xu Ma, Linzi Li, Hong Ding, Hexiao Shen, Yanli Hu, Xiaoxuan Zhu, Yonglian Zhang

**Affiliations:** 1 Jingmen Central Hospital, Jingmen Central Hospital Affiliated to Jingchu University of Technology, Hubei Clinical Medical Research Center for Functional Colorectal Diseases, Jingmen, Hubei, China; 2 School of Life Science, Hubei University, Wuhan, Hubei, China; 3 Maintainbiotech. Ltd., Wuhan, Hubei, China; 4 Department of Colorectal and Anal Surgery, Jingmen Central Hospital, Graduate Joint Training Base, School of Medicine, Wuhan University of Science and Technology, Jingmen, Hubei, China

**Keywords:** endurance exercise, gut microbiota, metagenomics, middle-aged adults, running

## Abstract

**Introduction:**

Exercise influences host metabolism and inflammation, but its functional effects on the gut microbiome in middle-aged populations remain unclear. This study used shotgun metagenomics to investigate the associations between long-term endurance running and the gut microbial ecosystem and its functional potential in middle-aged adults.

**Methods:**

We conducted a cross-sectional analysis comparing 33 middle-aged regular runners with 33 sedentary controls. No significant differences in age, BMI, dietary intake between groups. Fecal samples underwent metagenomic sequencing at an average depth of 10.97 Gb per sample. Following stringent quality control, taxonomic profiling, diversity analyses, and differential abundance testing were performed. Functional potential was annotated using GO, eggNOG, KEGG, CARD, VFDB, and CAZy databases.

**Results and discussion:**

The gut microbiota of middle-aged regular runners (RG, n = 33) and sedentary controls (CG, n = 33) was compared using metagenomic sequencing. No significant differences were observed between the two groups in terms of age, BMI, or self-reported dietary patterns. Although no significant differences in α-diversity or β-diversity were found, taxonomic profiling revealed differences in microbial community composition between the groups. Runners exhibited an increased relative abundance of carbohydrate-fermenting and short-chain fatty acid (SCFA)-producing species, including *Prevotella copri*, *Lachnospira eligens*, and *Collinsella intestinalis*. KEGG functional analysis revealed enrichment of genes associated with antibiotic biosynthesis pathways in runners, whereas the control group was enriched in genes related to lipid metabolism and xenobiotic degradation. The total abundance of antibiotic resistance genes (ARGs) and virulence factors (VFs) was significantly lower in runners. Carbohydrate-active enzyme (CAZy) profiling further indicated that runners harbored higher abundances of carbohydrate-binding modules and glycosyltransferase families, while controls were enriched in complex polysaccharide-degrading enzymes. Nonetheless, the cross-sectional design, qualitative dietary assessment, residual sex imbalance, and lack of metabolomic validation limit causal inference. Longitudinal intervention studies incorporating metabolomic analyses are warranted to confirm these associations and elucidate the directional adaptation of the gut microbiota to long-term regular exercise.

## 1 Introduction

The human gut microbiota comprises trillions of microorganisms inhabiting the gastrointestinal tract, forming a complex ecosystem whose collective genetic repertoire far surpasses that of the human genome. This microbiome has co-evolved with the host and plays essential roles in metabolism, immune regulation, and overall physiological homeostasis. Foundational studies have demonstrated that gut microbes contribute to nutrient processing, the production of short-chain fatty acids (SCFAs), and the modulation of intestinal barrier function, highlighting their critical role in host health and disease (Bäckhed et al., 2005; Ley et al., 2006; Yang et al., 2025). Increasingly, regular physical exercise is recognized as a key modulator of the gut microbiota, with growing evidence indicating that training volume, intensity, and athletic specialization exert distinct effects on microbial diversity and metabolic function (Mohr et al., 2020; Varghese et al., 2024).

Recent evidence from human exercise intervention studies has shown that physical activity can modulate the composition and function of the gut microbiota. High-intensity aerobic exercise over 9 weeks in healthy male college students increased the relative abundance of several bacterial genera, suggesting exercise-induced microbial shifts even in young, healthy adults (Donati Zeppa et al., 2021). Similarly, structured aerobic and resistance training over 6 months in overweight and obese adults led to alterations in microbial beta diversity and potential functional pathways, indicating that habitual exercise can modulate gut microbiota in populations at metabolic risk (Kern et al., 2020). Older adults also exhibit exercise-responsive microbiota changes. A 24-week combined cardiovascular and resistance program increased SCFA-producing genera such as Bifidobacterium, Oscillospira, and Anaerostipes, correlating with enhanced stool butyrate concentrations and metabolic improvements (Erlandson et al., 2021).

Current research on differences in gut microbiota between athletes and sedentary individuals exhibits heterogeneity. While some studies report no significant difference in α-diversity between the two groups, others suggest that gut microbial composition may vary depending on exercise types, training loads, and dietary patterns. Nevertheless, multiple studies indicate that long-term exercise training may be associated with the enrichment of specific microbial taxa in the gut and alterations in their functional characteristics. Specifically, athletes tend to have a higher abundance of microbial taxa involved in the production of short-chain fatty acids such as butyrate, lactate metabolism, vitamin biosynthesis, and anti-inflammatory functions, including taxa like *Prevotella* and *Akkermansia*. It should be noted, however, that the abundance of these microbial communities is also modulated by factors such as diet and host metabolic status (Clarke et al., 2014; Barton et al., 2018; Fontana et al., 2023; Sabater et al., 2023). Current research indicates notable interindividual differences in the association between gut microbiota and exercise. In elite athletes, metabolic pathways related to carbohydrate metabolism, amino acid synthesis, and short-chain fatty acid production are frequently enriched (Petersen et al., 2017; Cronin et al., 2018; O'Donovan et al., 2020). However, in sedentary populations, short-term exercise interventions have not been shown to induce significant changes in the functional metagenomic profile of the gut microbiota (Cronin et al., 2018). A hypothesis suggests that these adaptive changes may be linked to the host’s metabolic efficiency and physiological health, though the precise causal mechanisms remain to be fully elucidated (Fontana et al., 2023; Wosinska et al., 2024). It is noteworthy that the specific response patterns of the gut microbiota are significantly influenced by exercise modality, intensity, duration, and the athlete’s training level, distinguishing between elite and recreational athletes (Resende et al., 2021; Min et al., 2024). Furthermore, these studies indicate that in addition to exercise training itself, the distinct dietary patterns of athletes, such as high energy and protein intake, are also recognized as key confounding factors shaping these microbial traits. Research indicates that inadequate dietary fiber intake, when coupled with high protein consumption, may counteract the beneficial effects of exercise on gut microbial diversity (Son et al., 2020).

Despite considerable progress in athlete cohorts, far less is known about how habitual endurance exercise influences gut microbial function in middle-aged, non-professional populations. Prior work using 16S rRNA sequencing in middle-aged regular runners revealed significant differences in microbial composition and predicted functional pathways compared with sedentary peers (Duan et al., 2024). However, 16S rRNA sequencing has inherent limitations in characterizing the functional attributes of the gut microbiome. To gain genetic-level insights into how exercise influences the functional potential of the gut microbiome, this study utilized shotgun metagenomic sequencing to systematically compare the gut microbiota of middle-aged regular runners and sedentary controls. It is important to note that, similar to 16S sequencing, metagenomic sequencing primarily reveals the functional gene potential encoded by the microbial community, rather than its actual metabolic activity or *in situ* gene expression. As a cross-sectional observational investigation, it aimed to comprehensively map the compositional and functional profile of the gut microbiome in middle-aged habitual runners through high-resolution metagenomic analysis, identifying specific microbial taxa and functional features associated with long-term regular exercise. The findings are expected to provide a scientific basis for generating hypotheses and guiding future research into the underlying causal mechanisms.

## 2 Materials and methods

### 2.1 Participants

Running volunteers for this study were recruited from the Jingmen Long-Distance Running Association. Basic information, including age, gender, height, weight, resting heart rate, average weekly running distance (Km/week, recorded by KEEP APP), other lifestyle habits, and medical history, was collected through questionnaires. A survey on dietary intake was assessed using a Food Frequency Questionnaire (FFQ), taking into account a previous study by (Liang et al., 2019). The inclusion criteria were as follows:

1. Age range: 40–60 years old.
2. The average weekly running distance exceeded 40 km, and the duration of adherence was more than 1 year.
3. There was no hospitalization or need for parenteral nutrition or antibiotic treatment in the last 6 months.
4. Have not consumed excessive alcohol (≤ 15 g/per day) or psychoactive substances in the last 6 months.
5. Have not participated in a clinical trial in the last 6 months.
6. There were no cardiovascular, immune, gastrointestinal, or metabolic diseases.
7. Without specific dietary preferences.

Based on a cohort established in a previous 16S sequencing study (Duan et al., 2024) that included 25 regular runners and 22 healthy sedentary controls, the present study further recruited 8 eligible regular runners and 11 matched sedentary controls to enable a deeper metagenomic investigation. In total, 66 participants were included, comprising 33 regular runners and 33 sedentary controls. 33 healthy individuals with almost no exercise habits were selected from the medical examination department as the control group. They did not have a history of suspected gastrointestinal symptoms, cardiovascular disease, immune system disease, gastrointestinal disease, metabolic system disease, or any medication use in the last 6 months. They provided the exercise data for the most recent month by recall. Resting heart rate (RHR) was measured using an automated upper-arm blood pressure monitor (Omron HEM-7124, Omron Healthcare, China) after participants had rested in a seated position for at least 10 minutes. All measurements were performed in the morning under quiet conditions. The measurement was repeated twice with a 1-minute interval, and the average value was recorded for analysis.

All subjects volunteered to participate in the study and provided written informed consent. The study complied with the principles of the Declaration of Helsinki and was approved by the Ethics Committee of Jingmen Central Hospital.

### 2.2 Fecal sample collection and DNA extraction

All participants collected fecal samples at home using sterile fecal collection tubes, which consisted of a polystyrene plastic tube and a lid with a sampling spoon. The samples were then transported to the laboratory under dry ice storage. Each sample was divided into two 1.5-milliliter Eppendorf tubes and stored at -80 °C until DNA extraction. It should be noted that to avoid potential fluctuations in the microbial composition of the fecal samples before and after running, we required all participants to collect fecal samples in the morning. This is because running usually occurs in the afternoon or evening.

Genomic DNA was extracted using the HiPure Fecal DNA Mini Kit (Magen). DNA concentration was quantified using a Quantum Fluorometer (Thermo Fisher Scientific), and the integrity of the extracted genomic DNA was assessed via 1.5% agarose gel electrophoresis. Quality control standards included: DNA concentration ≥20 ng/μL, volume ≥20 μL, total amount ≥400 ng, with DNA integrity rated as good or slightly degraded; or DNA concentration between 5 and 20 ng/μL, volume ≥20 μL, total amount between 100 and 400 ng, with DNA integrity rated as good or slightly degraded.

### 2.3 Metagenomic sequencing

A double-end sequencing library with 300 bp insert fragments was constructed following the protocol of the TrueSeq Nano DNA Library Prep Kit (Illumina, USA). Library quality control was performed using an Agilent 2100 Bioanalyzer (Agilent Technologies, USA). Qualified libraries were sequenced on the Illumina Novaseq 6000 platform (Illumina, USA) to generate 2×150 bp paired-end reads. A total of 723.69 Gb of sequencing data were obtained from 66 fecal samples, averaging 10.97 Gb per sample, with Q30 ≥ 97.39%. Raw sequencing data were quality-assessed with FastQC v0.11.8 (Andrews, 2010), followed by read trimming using Trimmomatic v0.39 (Bolger et al., 2014). Within the KneadData v0.10.0 workflow (https://huttenhower.sph.harvard.edu/kneaddata/), cleaned reads were aligned with Bowtie2 v2.4.4 (Langmead and Salzberg, 2012) to remove host genomic sequences. Filtered reads were *de novo* assembled using MEGAHIT v1.2.9 (Li et al., 2015) with a minimum contig length threshold of 500 bp (**Supplementary Table 1**). Coding DNA sequences (CDS) were predicted from contiguous sequences using Prodigal v2.6.3, excluding genes shorter than 100 bp. CD-HIT v4.8.1 was employed to cluster genes across samples and eliminate redundancies (**Supplementary Table 2**). Salmon v1.10.1 was used to calculate transcript per million (TPM) values for each gene in all samples. TPM values were utilized as relative abundance metrics, with species and functional abundances derived by summing respective TPM values.

### 2.4 Taxonomic profiling

The resulting non-redundant gene set was aligned with the NCBI-NR protein database using DIAMOND v2.0.11 (Buchfink et al., 2015), and the alignment results were filtered based on an e-value threshold of ≤10^-^^5^. For the top 10 sequences ranked by score, the least common ancestor (LCA) method in MEGAN v6.24.20 software (Bağcı et al., 2021) was used to determine the taxonomic classification of each gene. The DIAMOND+NR+MEGAN pipeline was selected to achieve broader homology-based functional annotation capabilities, thereby supporting our exploratory analysis of non-core functions such as ARGs, VFs, and CAZy. The abundance of each taxon was calculated by summing the gene abundances assigned to that taxon.

### 2.5 Functional annotation of non-redundant genes

For functional annotation, the eggNOG-mapper v2.1.9 (Cantalapiedra et al., 2021) was used, with default parameters, to obtain non-redundant gene catalog annotations from the eggNOG (Huerta-Cepas et al., 2019) database. The Gene Ontology (GO) annotations were derived from the best matches in the NCBI-NR database and the “idmapping_selected.tab.gz” file from UniProt (updated version as of July 2022). KofamScan (exec_annotation script v1.3.0) (Aramaki et al., 2019) was used to perform KEGG annotation on the non-redundant gene set with an E-value threshold of 1e-5. CAZyme annotation was conducted using dbcan3 v3.0.1 (Zhang et al., 2018) with default parameters. Antibiotic resistance gene (ARG) annotation was performed using rgi v6.0.1 in conjunction with the CARD database (Alcock et al., 2023). To identify virulence factors, blastp v2.5.0+ was used to search the VFDB Core DNA Sequence Collection (Liu et al., 2018), selecting the highest-scoring match (**Supplementary Table 3**).

### 2.6 Statistical analysis

To comprehensively characterize the structure and functional profiles of the gut microbiome, we employed a series of statistical analyses. α-Diversity indices for each sample were calculated using QIIME 2 (Bolyen et al., 2019) and the vegan package in R v4.3.1 (R., 2023). The Wilcoxon rank-sum test was used to evaluate the differences in α-diversity between the two groups. β-Diversity at both the taxonomic (species) and functional levels was evaluated using principal coordinate analysis (PCoA). To assess the overall differences in microbial community structure between groups, permutational multivariate analysis of variance (PERMANOVA) was performed based on Bray-Curtis distance matrices. Additionally, analysis of similarities (ANOSIM)​was used as a non-parametric supplementary test. For differential testing of taxonomic abundances and functional profiles, we employed the Wilcoxon rank-sum test, In the data analysis, to control for the influence of gender, aligned rank transform analysis of variance (ART ANOVA) was applied to examine species composition, KOs, CAZy, ARGs, and virulence factors, with gender incorporated as a covariate in the model. The analysis was conducted using the ARTool package (v0.11.2) in R (v4.3.1), with a statistical significance threshold of *p* < 0.05. Additionally provided the results adjusted by the Benjamini-Hochberg false discovery rate (FDR) method for reference (**Supplementary Tables 4**-**S12**). To identify key microbial taxa and functional pathways that differed across groups, linear discriminant analysis effect size (LEfSe) was applied for differential abundance analysis, with an LDA score > 2 used as the threshold for biologically meaningful features. Spearman’s rank correlation analysis was conducted to assess associations between microbial species and KEGG pathways, and significant correlations were visualized using clustered heatmaps. All statistical analyses and visualizations were performed in R, primarily using the ggplot2 v3.5.2 and ggalluvial v0.12.5 packages (Wickham, 2016; Brunson and QD, 2024).

## 3 Results

### 3.1 Baseline population characteristics

A total of 66 fecal samples were included in this study (Runner = 33, Control = 33). The demographic data are presented in **Table 1**. After controlling for sex, analysis of covariance revealed statistically significant differences between the runner and control groups in heart rate and weekly running mileage (p < 0.001, effect size η² > 0.75), but not in age or BMI (**Supplementary Table 13**). Moreover, comparison of dietary structure data based on the Food Frequency Questionnaire showed no significant difference in overall dietary patterns between the two groups.

**Table 1:** Baseline characteristics and dietary habits of participants in the RG and CG groups.

Variables	RG (N = 33)	CG (N = 33)	P	Statistical test
Gender(Male/Female)	21/12	8/25	0.0029 (**)	Chi-square
Age	49.42 ± 3.17	49.27 ± 3.69	0.8587 (ns)	Welch t-test
BMI	22.75 ± 1.88	23.04 ± 3.71	0.626 (ns)	Mann-Whitney U
Heart rate (beats/min)	57.70 ± 4.25	73.24 ± 4.17	<0.001 (***)	Mann-Whitney U
Km/week	45.43 ± 2.65	3.77 ± 1.69	<0.001 (***)	Welch t-test
Dietary Habits (n)				
Dairy products (Fr/S/O/N)	9/8/11/5	8/12/7/6	0.8785 (ns)	Mann-Whitney U
Fried and fatty food (Fr/S/O/N)	3/4/9/17	3/5/12/13	0.4254 (ns)	Mann-Whitney U
Refined carbohydrates (Fr/S/O/N)	4/2/23/4	1/5/24/3	0.9487 (ns)	Mann-Whitney U
High protein foods (Fr/S/O/N)	32/1/0/0	28/3/2/0	0.6857 (ns)	Mann-Whitney U
Fruits and vegetables (Fr/S/O/N)	29/4/0/0	27/5/1/0	0.4768 (ns)	Mann-Whitney U
Protein supplementation (Fr/S/O/N)	1/7/9/16	0/8/9/16	0.9613 (ns)	Mann-Whitney U

**p < 0.01, ***p < 0.001.

### 3.2 Diversity profiling of the gut microbiome

**Figure 1:**
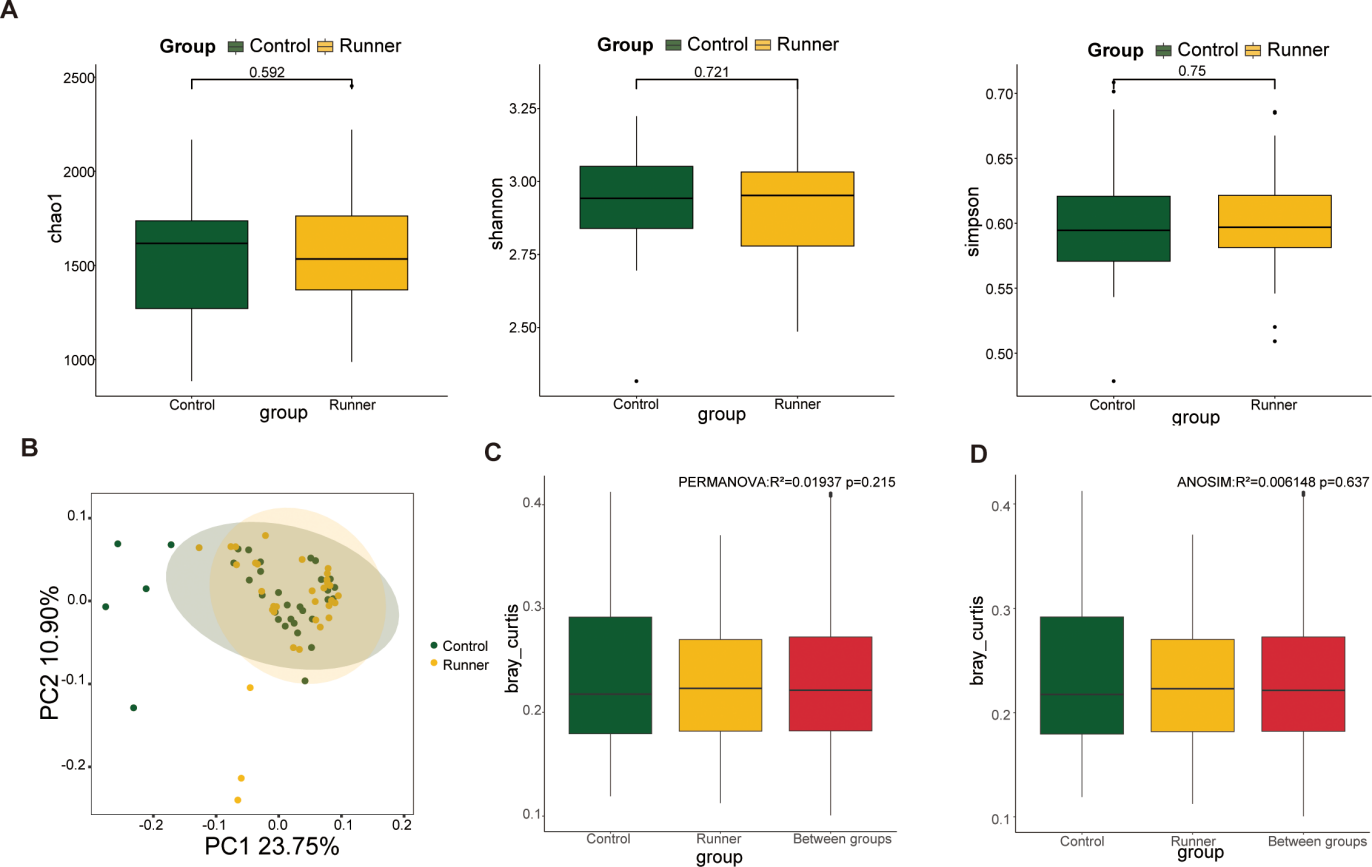
α-diversity and β-diversity analysis of gut microbiota between CG and RG groups. **(A)** Comparison of α-diversity indices (Chao1, Shannon, and Simpson) between CG (green) and RG (yellow) groups. No significant differences were observed in Chao1, Shannon diversity, or Simpson diversity. Statistical significance was determined by Wilcoxon rank-sum test. **(B)** Principal Coordinate Analysis (PCoA) based on Bray-Curtis distance showing the overall microbial community structure. Each point represents an individual sample, with ellipses indicating 95% confidence intervals. **(C)** Boxplot of PERMANOVA analysis based on Bray-Curtis distance. **(D)** Boxplot of ANOSIM analysis based on Bray-Curtis distance.

Diversity analysis of the gut microbiota species composition was conducted using sequencing data from 66 fecal samples, comparing the RG and CG groups. α-Diversity, as assessed by the Chao 1, Shannon, and Simpson indices, showed no significant differences between the RG and CG groups (**Figure 1A**). β-Diversity was evaluated using principal coordinate analysis (PCoA) based on Bray-Curtis (**Figure 1B**). Both PERMANOVA and ANOSIM analyses indicated no significant differences in the overall gut microbiota structure between the two groups (**Figures 1C, D**).

### 3.3 Analysis of gut microbial structure differences

**Figure 2:**
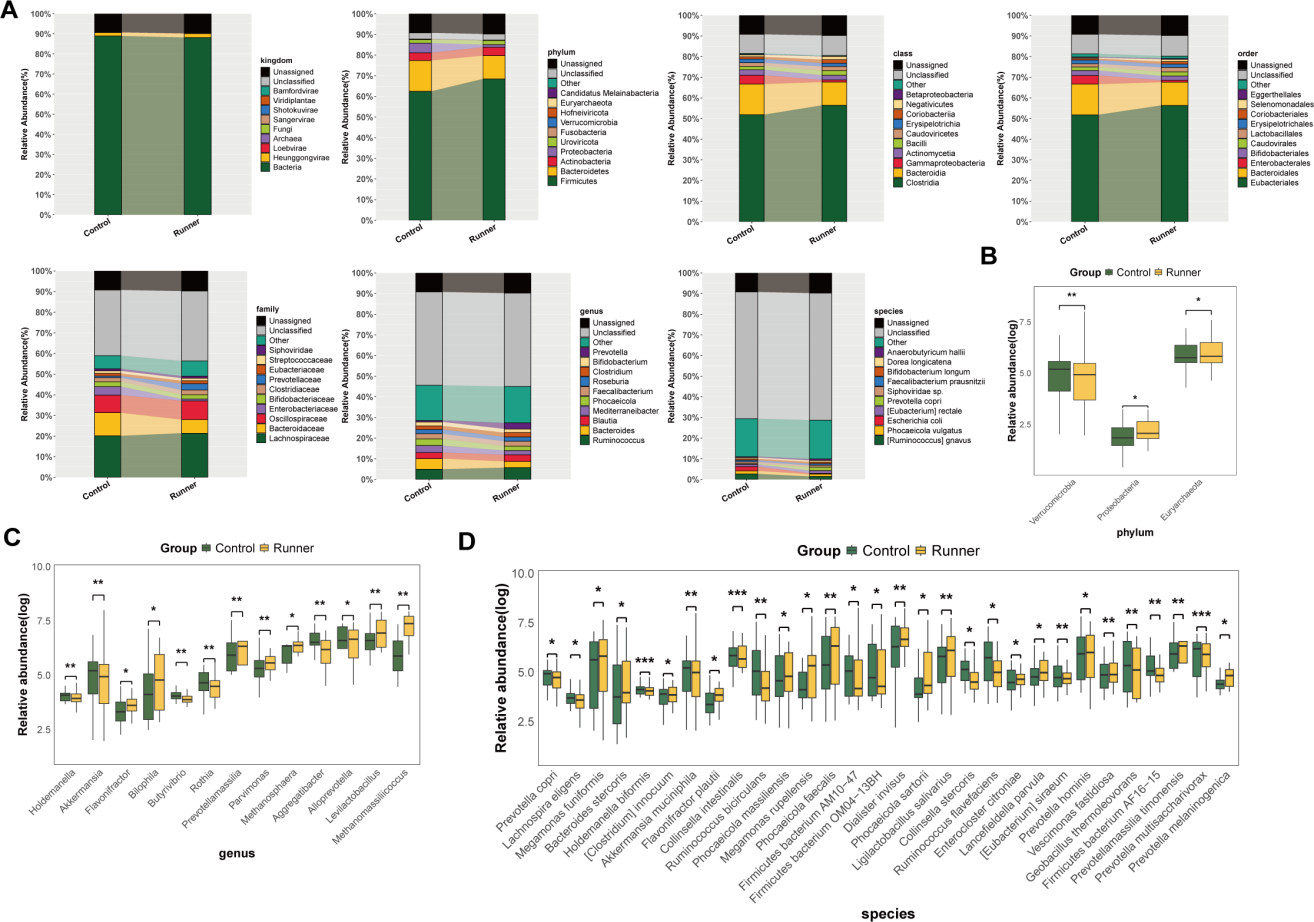
Taxonomic composition and differential abundance analysis of gut microbiota at multiple levels. **(A)** Relative abundance of microbial taxonomic groups at the phylum, class, order, family, genus, and species levels. The stacked bar chart shows the average relative abundance of top taxonomic groups in the CG and RG. **(B–D)** Box plots show the relative abundance (logarithmic transformation) of differential abundance taxonomic groups at the phylum **(B)**, genus **(C)**, and species **(D)** levels. Significant differences were determined by the ART ANOVA analysis for gender covariates. *p < 0.05, **p < 0.01, ***p < 0.001.

Based on the NR database, microbial composition was profiled across taxonomic levels: phylum, class, order, family, genus, and species (**Figure 2A**, **Supplementary Table 14**). Gender-covariate analysis revealed that at the phylum level, *Proteobacteria* and *Euryarchaeota* were significantly enriched in the RG, while the relative abundance of *Verrucomicrobia* was significantly lower compared to the CG (**Figure 2B**). At the genus level, three genera, *Butyrivibrio*, *Rothia*, and *Aggregatibacter* were significantly enriched in RG, whereas the other nine significantly different genera were enriched in CG, including *Flavonifractor*, *Bilophila*, and two methanogenic archaeal genera. (**Figure 2C**). At the species level, species significantly enriched in RG included *Prevotella copri*, *Lachnospira eligens*, *Megamonas funiformis*, *Collinsella intestinalis*, and *Ruminococcus bicirculans*, which are primarily involved in carbohydrate fermentation and short-chain fatty acid production. In contrast, species significantly enriched in CG included *Bacteroides stercoris*, *Akkermansia muciniphila*, and *Phocaeicola sartorii* (**Figure 2D**).

### 3.4 Functional annotation analysis of gut microbiomes with GO, EggNOG and KEGG

**Figure 3:**
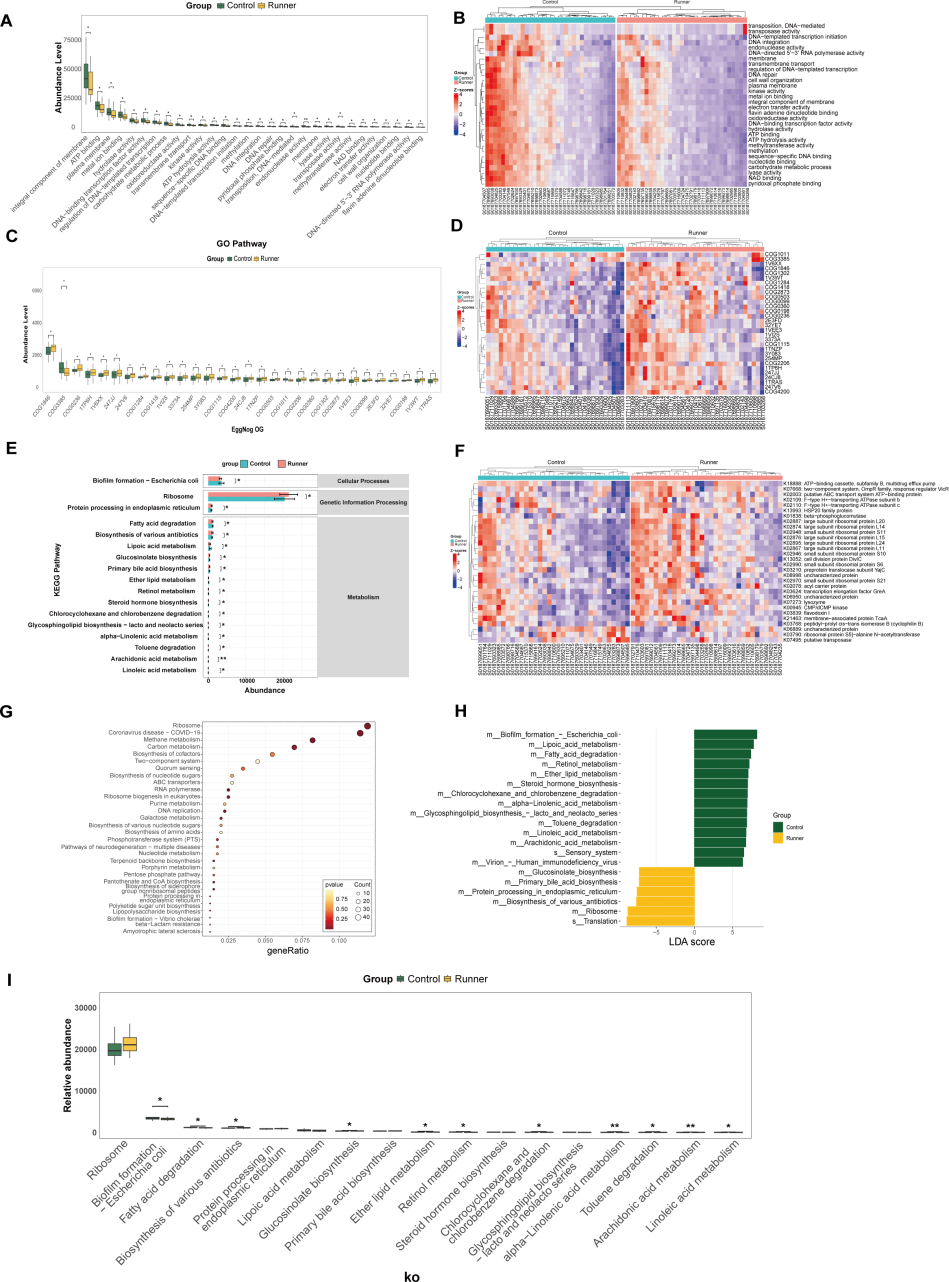
Functional profiling and pathway enrichment analysis based on GO, eggNOG, and KEGG database. **(A)** Boxplot showing differentia l analysis of the top 30 significantly different Gene Ontology (GO) terms. Boxplots display the abundance and significance of GO terms in the RG and CG groups. **(B)** Heatmap of differentially abundant GO terms. The color scale represents Z-score normalized abundance (red: high, blue: low) with hierarchical clustering. **(C)** Relative abundance of the top 30 significantly different EggNOG (OG) terms between the CG and RG groups. **(D)** Heatmap of differentially abundant EggNOG terms. The color scale represents Z-score normalized abundance (red: high, blue: low) with hierarchical clustering. **(E)** Relative abundance of the top 30 significantly different KEGG level 1 pathways and KO pathways between the CG and RG groups. Level 1 pathways are categorized into Metabolism, Genetic Information Processing, and Cellular Processes. **(F)** Heatmap of differentially abundant KEGG KO modules (red: high, blue: low). **(G)** Bubble plot showing enriched KEGG pathways based on gene ratio and P-value. Dot size represents gene count, and color intensity represents significance level. **(H)** LEfSe plot showing differentially abundant KEGG modules between groups (LDA score > 2.0). **(I)** The boxplot shows the results of the ART ANOVA differential analysis for gender covariates on the top 30 significantly different KOs.*p < 0.05, **p < 0.01, ***p < 0.001.

GO functional enrichment analysis, based on relative gene abundance, revealed distinct patterns between the RG and CG groups (**Supplementary Table 15**). Among the top 30 most significantly different terms (based on nominal p-value < 0.05), those showing significantly lower abundance in the RG were predominantly associated with membrane systems and energy metabolism. Specifically, membrane-related components and activities, including integral membrane components, plasma membrane, ATP binding, metal ion binding, hydrolase activity, oxidoreductase activity, and transmembrane transport, were all present at significantly lower abundance in the RG compared to the CG. Terms related to transcriptional regulation, such as DNA-binding transcription factor activity and DNA-templated transcription, also showed lower gene abundance. Similarly, terms associated with carbohydrate metabolism and kinase activity were also decreased. Moreover, functions linked to genome maintenance, including ATP hydrolysis, sequence-specific DNA binding, DNA repair, DNA integration, and transposase activity, demonstrated significantly lower relative gene abundance in RG (**Figures 3A, B**).

Based on eggNOG functional annotation analysis, among the top 30 function categories with significant differences (based on nominal p-value < 0.05), the RG showed significantly higher relative gene abundance compared to CG in ABC transport systems, DNA-binding transcription factors, mRNA degradation proteins, and fatty acid synthesis–related modules. In contrast, transposase-related functions exhibited significantly lower relative gene abundance in RG (**Figures 3C, D**).

The KEGG enrichment analysis results showed that compared with the CG, the RG exhibited an overall higher relative abundance of genes in key KEGG pathways (**Supplementary Tables 16**, **S17**). Among the KO genes with significant differences and top 30 abundance, 27 genes showed higher abundance and three genes showed lower abundance (**Figures 3E, F**). Genes of higher abundance were predominantly associated with ABC transport systems, ribosomal protein synthesis, and stress-response pathways. Membrane-related proteins, including TcaA, ATP-binding proteins of the ABC transport system, and F-type H^+^-transporter ATPase, which are associated with membrane transport and energy metabolism, presented higher abundance. The KEGG enrichment bubble plot (**Figure 3G**) indicated that the five most significantly enriched pathways in the RG were ribosome, coronavirus disease–COVID-19, methane metabolism, carbon metabolism, and biosynthesis of cofactors. The LEfSe analysis revealed that the RG specifically enriched pathways such as translation, ribosome, and primary bile acid biosynthesis, while the CG enriched pathways such as fatty acid degradation and arachidonic acid metabolism (**Figure 3H**).

Gender-covariate analysis revealed that the core functional profile remained robust. The RG maintained significantly higher relative gene abundance in pathways responsible for the biosynthesis of various antibiotics. Conversely, the CG demonstrated consistently elevated relative gene abundance in pathways associated with lipid metabolism, such as arachidonic acid, α-linolenic acid, and fatty acid metabolism. Significant increases were also observed in pathways for xenobiotic degradation, including toluene, chlorocyclohexane, and chlorobenzene degradation, as well as in those linked to Escherichia coli biofilm formation (**Figure 3I**).

### 3.5 Resistance gene annotation analysis and virulence profiling of gut microbiomes

**Figure 4:**
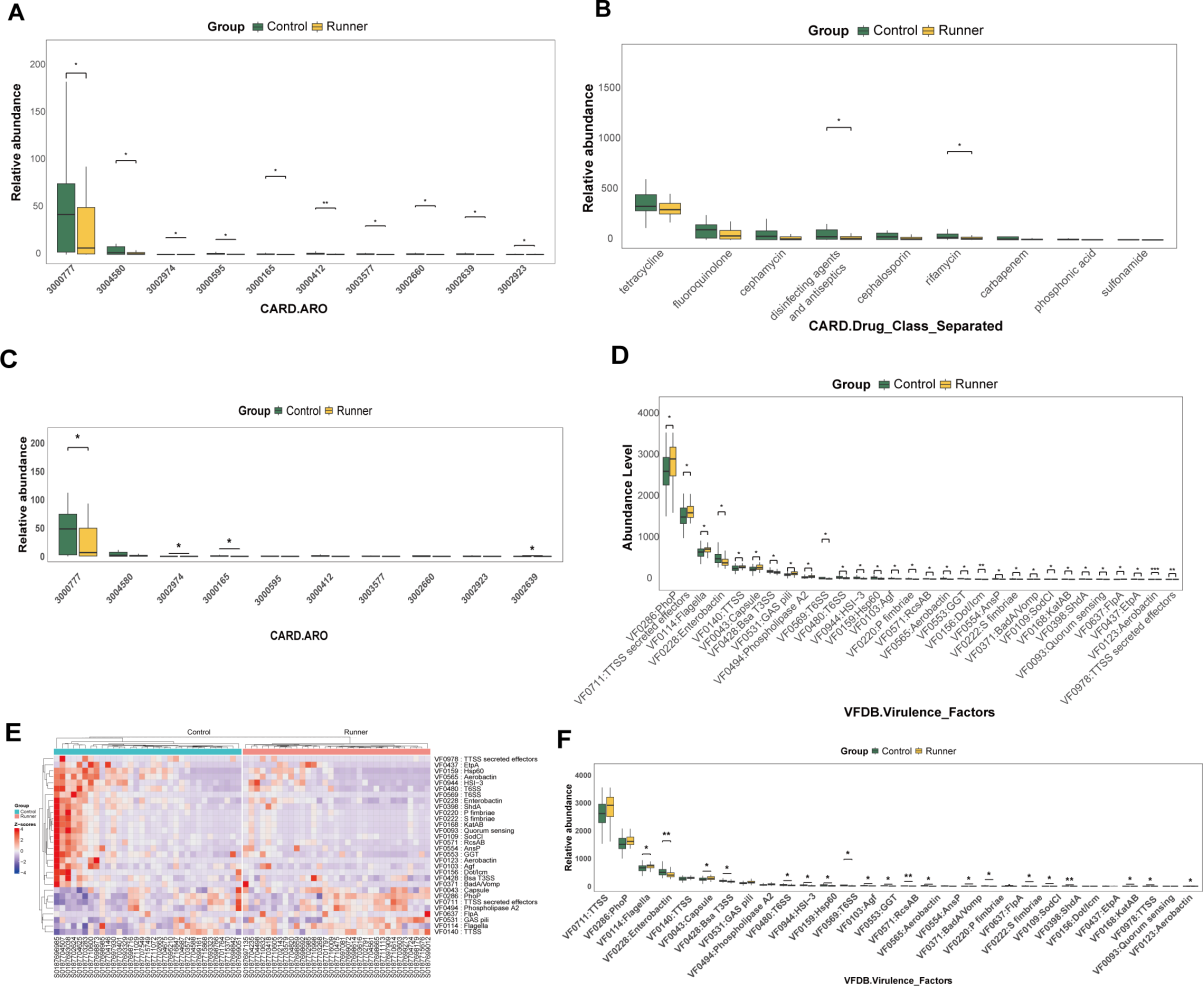
Analysis of antibiotic resistance genes (ARGs) and virulence factors. **(A)** Relative abundance of antibiotic resistance genes (ARGs) annotated by the CARD database at the ARO (Antibiotic Resistance Ontology) level. **(B)** Relative abundance of ARGs categorized by drug class. **(C)** Boxplot of ARGs relative abundance differences from ART ANOVA analysis with gender covariates. **(D)** Relative abundance of virulence factors annotated by the VFDB (Virulence Factor Database). Boxplots display the abundance of major virulence factors. **(E)** Heatmap showing Z-score normalized abundance of individual virulence factors across samples with hierarchical clustering. Statistical significance was determined by Wilcoxon rank-sum test (red: high, blue: low). **(F)** Boxplot of virulence factors relative abundance differences from ART ANOVA analysis with gender covariates.*p < 0.05, **p < 0.01, ***p < 0.001.

The results of the CARD database profiling showed that there were significant differences in 10 resistance genes between the two groups (**Supplementary Table 18**). Among them, 9 genes showed significantly lower abundance in the RG group (**Figure 4A**). The total abundance of antibiotic resistance genes in the intestinal microbiota of the RG was significantly lower than that of the CG (**Figure 4B**). Following gender-covariate adjustment, the genes that remained significantly lower in abundance were restricted to RND efflux pumps and target-modification-related resistance genes vanR and Erm (**Figure 4C**).

Based on the VFDB database, the abundance differences of virulence factor–related genes in the intestinal microbiota between long-term endurance-trained individuals and sedentary controls were analyzed (**Supplementary Table 19**). Among the 508 virulence factors tested, 31 differed significantly in abundance between RG and CG (**Figures 4D, E**). Following gender-covariate adjustment, the RG consistently exhibited lower relative abundance of several well-characterized virulence factors, including the iron acquisition systems aerobactin and enterobactin, the adhesion factors P fimbriae, S fimbriae, and ShdA, and the type VI secretion system. In contrast, the capsule biosynthesis factor showed a significantly higher abundance in the RG (**Figure 4F**).

### 3.6 Carbohydrate active enzyme profiling of gut microbiomes

**Figure 5:**
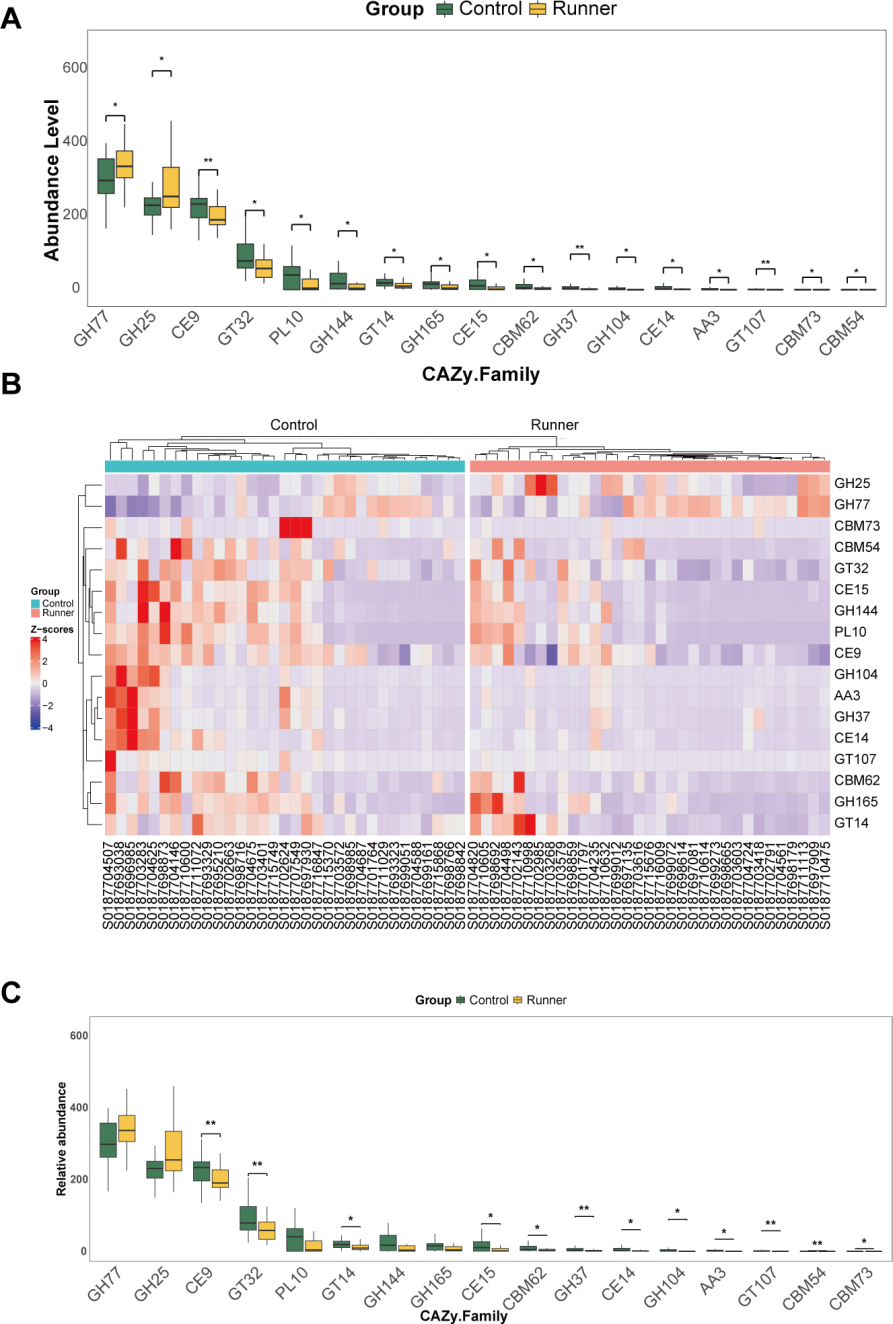
Carbohydrate-active enzymes (CAZy) profiling analysis. **(A)** Relative abundance of CAZy families in the CG and RG groups. Boxplots show the abundance distribution of major CAZy families including Glycoside Hydrolases (GH), Glycosyl Transferases (GT), Carbohydrate Esterases (CE), Polysaccharide Lyases (PL), Auxiliary Activities (AA), and Carbohydrate-Binding Modules (CBM). **(B)** Heatmap showing Z-score normalized abundance of individual CAZy families across samples with hierarchical clustering (red: high, blue: low). **(C)** Boxplot of CAZy families relative abundance differences from ART ANOVA analysis with gender covariates. *p < 0.05, **p < 0.01, ***p < 0.001.

Among the 282 CAZy families, 17 differed significantly in relative abundance between the RG and the CG (**Supplementary Table 20**). Specifically, the relative abundance of 15 families were significantly lower in the RG (**Figures 5A, B**). Following sex−stratified adjustment, families that remained robustly lower in the RG were functionally annotated to roles in complex polysaccharide degradation (GH43, GH92, GH2), bacterial cell wall metabolism (GH104, GH25, CE9, CE14), glycan synthesis and modification (GT107, GT32, GT14), and carbohydrate binding (CBM73, CBM62, CBM54). In contrast, families that remained significantly higher in the RG after sex−stratified adjustment were primarily annotated to functions associated with carbohydrate−binding module (CBM2) and glycosyl transferase (GT31, GT76) (**Figure 5C**).

## 4 Discussion

This study systematically evaluated the impact of long-term endurance running on the gut microbiota of middle-aged regular runners using metagenomic sequencing. The results indicated that, although no significant changes were observed in α-diversity or β-diversity, the gut microbiome of runners displayed distinct compositional and functional profiles compared to sedentary controls. Specifically, runners had a higher relative abundance of bacterial taxa associated with short-chain fatty acid-production and a lower relative abundance of some taxa often linked to opportunistic pathogenesis. Moreover, genes related to energy transport and protein synthesis were also more abundant in the RG.

Regarding species composition, the findings of the present study differ from our previous 16S rRNA analysis (Duan et al., 2024), which reported significant β-diversity differences. This discrepancy may be explained by the combined effects of an expanded and demographically distinct cohort, the higher analytical resolution of shotgun metagenomics, and the statistical control for sex applied in the current study. Consequently, this study did not find significant differences in α- or β-diversity between the RG and CG groups. The lack of significant diversity changes aligns with multiple intervention studies. For instance, a 24-week exercise trial in young sedentary adults found no significant effects of different training intensities on α-diversity or β-diversity (Martinez-Tellez et al., 2025). Similarly, a systematic review in elderly populations reported that most exercise interventions did not significantly alter diversity indices (Aya et al., 2023). Collectively, these findings suggest that exercise primarily affects specific taxonomic groups and functional capacities rather than overall microbial community diversity.

From a physiological perspective, the observed differences in gut microbial composition and functional potential may have biological implications. Long-term exercise has been shown to modulate microbial composition and increase the production of metabolites such as short-chain fatty acids, which provide energy to intestinal epithelial cells and regulate systemic metabolism and inflammatory responses (Monda et al., 2017; Blaak et al., 2020; Aya et al., 2021; Dziewiecka et al., 2022; Onu et al., 2026). At the species level, *Prevotella copri*, which was significantly enriched in the RG group, is strongly associated with carbohydrate fermentation and short-chain fatty acid production. As a dominant species in the gut of athletes, the abundance of *P. copri* shows a significant positive correlation with weekly exercise duration and the number of carbohydrate and amino acid metabolic pathways, maintaining the highest abundance in endurance athletes. Through dietary fiber fermentation, it produces SCFAs such as propionate and valerate, providing energy substrates for prolonged exercise (Petersen et al., 2017; Mohr et al., 2020). However, enrichment of *P. copri* may also come with potential metabolic costs. Nieman et al. (2025) found a strong positive correlation between intestinal *P. copri* abundance and pro-inflammatory oxidative lipids (ARA-CYP oxylipins) post-exercise, with approximately two-thirds of the variance in post-exercise inflammation explainable by this single species. This suggests that athletes with high *P. copri* abundance may exhibit more pronounced inflammatory responses after high-intensity exercise (Nieman et al., 2025). In addition, we observed that the CG group showed significant enrichment of *Akkermansia* (AKK), which contrasts with most current studies reporting a notable increase in AKK abundance following exercise. One possible explanation is that the *Prevotella*-dominated fermentative niche enriched in the RG group may create ecological niche competition against AKK. Another plausible reason is that subtle differences in dietary patterns could remain a potential confounding factor. Although no significant overall dietary pattern differences were detected between the two groups in this study, Clarke et al. (2014) pointed out that discrepancies in total energy, protein, and dietary fiber intake between athletes and sedentary controls are important covariates shaping the gut microbiota (Clarke et al., 2014). High protein intake is generally negatively correlated with AKK abundance, and the protein requirements of long-distance endurance runners are often higher than those of sedentary individuals. Subtle shifts in macronutrient proportions could also influence the colonization of AKK.

Functional annotation analyses further revealed differences in the microbial functional gene repertoire between groups. GO annotation indicated a lower relative abundance of genes​associated with basic cellular maintenance functions in the RG, including those related to membrane processes, ATP binding and hydrolysis, oxidoreductase activity, and transmembrane transport. This pattern, together with the observed lower abundance of transposase activity genes in the eggNOG analysis, may suggest a potentially higher genomic stability and a relatively lower allocation of metabolic resources towards basal maintenance in the gut microbiota of runners. Concurrently, KEGG pathway analysis demonstrated a lower abundance of genes associated with the degradation of exogenous compounds. This functional signature aligns with observations in elite athlete cohorts, wherein the gut microbiota exhibits a metabolic reorientation, shifting priority toward pathways for amino acid and carbohydrate metabolism and energy production, and away from basal maintenance and xenobiotic processing (Barton et al., 2018).

In contrast to the lower abundance of genes for these basic functions, eggNOG annotation indicated a higher abundance of genes associated with ABC transport systems and fatty acid synthesis modules in the RG. Meanwhile, CAZy annotation revealed a lower abundance of enzyme families linked to complex polysaccharide degradation, bacterial cell wall metabolism, and glycosylation. Consequently, the functional gene abundance profile observed in the RG is distinguished by a general increase in genes associated with nutrient transport, energy transduction, and biosynthesis, coupled with a decrease in those dedicated to basal maintenance. This profile aligns with the functional signatures observed in the gut microbiota of athletes. Previous studies have similarly reported that metabolic and energy-related pathways are more prominent in the microbiomes of physically active individuals compared with sedentary controls (Petersen et al., 2017; Allen et al., 2018; Munukka et al., 2018; Chen and Vitetta, 2020; Fontana et al., 2023; Varghese et al., 2024).

Furthermore, analysis of antibiotic resistance genes (ARGs) and virulence factors (VFs) extended the functional disparities described above. The overall relative abundance of both ARGs and VFs was lower in the RG compared to the CG. Specifically, the RG exhibited a lower abundance of several ARG classes, including RND efflux pumps and target site modification-related resistance genes (vanR and Erm). Regarding VFs, genes associated with iron acquisition and bacterial adhesion, which are functions often linked to host colonization and immune evasion, were also less abundant in the RG. Although a slight increase was observed for certain toxin- and protease-related genes, the overall abundance of genes for secretion systems and iron acquisition pathways remained lower. Together, the lower abundance of ARG- and VF-related genes constitutes another feature of the functional gene profile in the RG. This feature aligns with observations in physically active populations, whose gut microbiomes tend to harbor a lower burden of ARGs and reduced abundances of some potentially pathogenic bacteria (Clarke et al., 2014; Allen et al., 2018).

In conclusion, our shotgun metagenomic analysis reveals that long-term endurance running in middle-aged adults is significantly associated with a distinct functional potential of the gut microbiome, without accompanying changes in overall microbial community diversity. This functional signature is characterized by the enrichment of genes related to nutrient transport, energy conversion, and biosynthetic processes, alongside lower abundances of genes involved in basic cellular maintenance, antimicrobial resistance, and virulence.

However, several important limitations should be noted. In this study, nominal p-values were used in the differential abundance analysis to enhances statistical power to detect potential associations, though this approach may increase the potential for false positive findings. Therefore, we have provided q-values (FDR) in the supplementary materials for reference. While dietary intake is a primary determinant of gut microbiome composition, yet we were only able to obtain qualitative dietary pattern data based on a food frequency questionnaire, without detailed quantitative nutrient information. Consequently, diet represents a significant and unmeasured confounding factor. Similarly, although we statistically adjusted for sex, a significant sex imbalance between the groups persists, which may introduce residual confounding. With regard to physical activity, methods for assessing its exposure were inconsistent between the groups, and detailed exercise intensity data were unavailable. Therefore, the possibility of residual confounding cannot be entirely ruled out and should be considered when interpreting the results. Importantly, potential variability introduced by stool sample collection represents an additional limitation, as neither the precise timing of collection nor stool consistency was controlled for or recorded. Moreover, the metagenomic sequencing data reflect the functional potential of the microbiota rather than direct evidence of host physiological status, and the study cohort consisted of a specific middle-aged population, which calls for caution in generalizing the findings. Due to the cross-sectional observational design of this study, a causal relationship underlying the observed associations cannot be inferred. Future longitudinal or intervention studies incorporating metabolomic approaches are warranted to validate these observed associations and clarify the directionality of microbiota changes.

## Data Availability

The raw sequence data generated in this study are deposited in the NCBI Sequence Read Archive (SRA) under BioProject accession number PRJNA1457655.
